# Recent progress in biochar-supported photocatalysts: synthesis, role of biochar, and applications

**DOI:** 10.1039/c8ra02258e

**Published:** 2018-04-17

**Authors:** Md Manik Mian, Guijian Liu

**Affiliations:** CAS Key Laboratory of Crust-Mantle Materials and Environments, School of Earth and Space Sciences, University of Science and Technology of China Hefei 230026 China lgj@ustc.edu.cn +86 551 63621485 +86 551 63603714; State Key Laboratory of Loess and Quaternary Geology, Institute of Earth Environment, The Chinese Academy of Sciences Xi’an Shaanxi 710075 PR China

## Abstract

Incorporating photocatalytic nanoparticles with biochar templates can produce biochar-supported photocatalysts (BSPs) and combine the advantages of biochar with catalytic nanoparticles. The obtained composite exhibits excellent surface properties, crystallinity, chemical stability, recoverability, and higher photocatalytic competency than the bare semiconductor photocatalyst. The literature and advances in BSPs based on the combination of low-cost biochar and catalytic nanoparticles are presented in this review. Various synthetic techniques and physicochemical properties of BSPs are summarized. The article then discusses in detail the important role of biochar in influencing the photocatalytic performance of BSPs such as supporting nanoparticles, increasing the surface area and the number of active sites, shuttling electrons, acting as an electron reservoir, increasing charge separation, and reducing band gap energy. Furthermore, the synergistic effects of adsorption and photodegradation of organic pollutants by BSPs are discussed with in-depth mechanistic evidence. Finally, the application of BSPs in various fields and constructive suggestions for their future development are reported.

## Abstract 2

Incorporating photocatalytic nanoparticles with biochar templates can produce biochar-supported photocatalysts (BSPs) and combine the advantages of biochar with catalytic nanoparticles.

## Graphical abstract

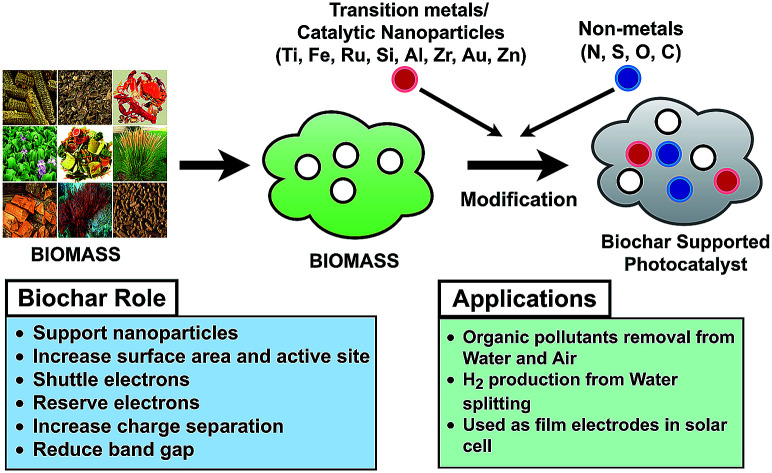

## 1. Introduction

Nowadays, environmental pollution and insufficient sources of natural clean energy are serious global problems faced by humankind. The high growth rate of the world’s population, industrialization, and urbanization accelerates the consumption of non-renewable energy and causes the release of pollution into the air and waterways. According to a UN water report, on average 70%, 28–38%, and 8% municipal wastewater is treated before discharge in high-income, middle-income, and low-income countries, respectively, and about 80% of total global wastewater is discharged into the environment without any treatment.^1^ Untreated or improperly treated wastewater discharges certainly impose a detrimental effect on the natural ecosystem and public health. Therefore, a diverse group of researchers devoted themselves to find an environmentally friendly, cost-effective, and sustainable way to resolve this adverse situation. From that perspective, photocatalysis, in which naturally available, safe, and clean solar energy has been utilized by semiconductor photocatalysts to degrade harmful organic pollutants, is a major advancement.

Up until now, various nanoparticles such as RuO_2_, TiO_2_, Fe_2_O_3_, SiO_2_, Al_2_O_3_, ZrO_2_, ZnO, CdS, and ZnS have been utilized as semiconductor photocatalysts.^2–6^ Among them, different forms of TiO_2_ nanoparticles have received significant attention due to their chemical stability, low toxicity, inexpensiveness, availability, and high sensitivity to UV light illumination.^7^ However, the large band gap energy and the separation of TiO_2_ nanoparticles are the major drawbacks for their applications. Furthermore, TiO_2_ cannot utilize most of the solar energy spectrum due to its large band gap energy.^4^ Therefore, an external UV light source is required for their activation, which is not a cost-effective method. However, various hybrid composites synthesized by combining doping of transition metals (Ti, Fe, Ru, Au, and Mo)^8–11^ and/or non-metals (C, N, and S)^12–14^ can improve the photocatalytic performance, but, high preparation costs, non-renewability, and risks of secondary pollution prevent their large-scale application. Therefore, more and more attention has been applied to giving a stable platform for photocatalytic nanoparticles by incorporating secondary materials, which can improve the recoverability and visible light sensitivity of the photocatalysts. Biochar, as a supporting material for catalytic nanoparticles, receives significant attention in this direction. The unique physicochemical properties of biochar are beneficial for incorporating various photocatalytic nanoparticles.^2,15^

**Fig. 1:**
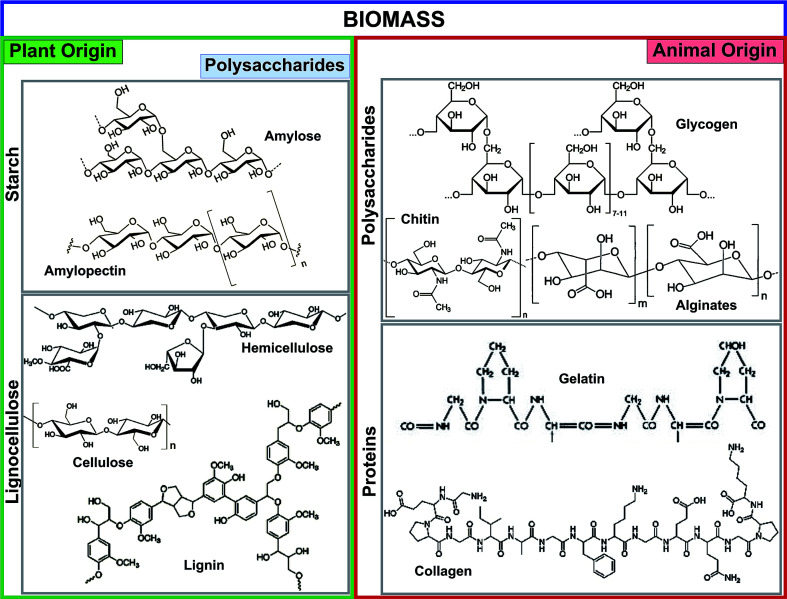
Prominent fractions of biomass feedstocks derived from plants and animals.

Biochar is a low-cost, stable, environmentally friendly, and sustainable material produced from available waste biomass *via* pyrolysis, hydrolysis, gasification, and carbonization methods.^16,17^ The prominent fractions of plant- and animal-originated biomass used for biochar production are illustrated in Fig. 1. The major streams of biomass are lignocellulose, starch, animal protein, and polysaccharides. Starch and lignocellulose are the forms of plant-driven biomass used for the synthesis of biochar and different chemicals. Starch is composed of a large number of glucose units connected by glycosidic bonds and consists of two molecules such as amylose and amylopectin. Starch is commonly used as a pore-forming agent in ceramic technology.^18^ The composition of lignocellulose, especially lignin and hemicellulose structures, is highly dependent on its origins such as hardwood, softwood, or grasses. Lignin is a constituent of the cell wall, and is the second most abundant polymer after cellulose. Lignin, composed of three different phenyl propane monomers, is electronegative and has a strong affinity for various electropositive metal ions.^2^ Among animal-originated polysaccharides, chitin is the most abundant natural polysaccharide, and can be found in the exoskeleton of insects and crustacean cell walls.^19^ The amino (chitin, chitosan, gelatin, and collagen) and hydroxyl (chitin, chitosan, and glycogen) functional groups of animal-origin biochar possess strong adsorption capacities for various heavy metals,^20^ dyestuffs,^20^ and pathogens.^21^ Moreover, the –OH and –NH_2_ functional groups of biomass can easily be tuned with other functional materials.

Biochar can be used as an excellent platform for supporting various catalytic nanoparticles due to its unique surface properties, easily tunable functional groups, chemical stability, and electrical conductivity.^22–24^ The electron-conductive nature of biochar can reduce the quick recombination of the e^−^/h^+^ pair during photocatalysis^25^ and suitable surface functional groups enable immobilization of different pollutants, which is favorable for photocatalysis.^26,27^ An excellent review article published recently describes the TiO_2_-immobilized carbon from different renewable sources and introduces the application of biochar in the synthesis of photocatalysts as well.^2^ Consequently, various biochar-supported photocatalysts have been synthesized and reported in recent years. The reported BSPs show promising improvements in visible light sensitivity, chemical stability, recollection ability, and photocatalytic degradation of various pollutants. Some of those recently reported BSPs are TiO_2_–SPW, TiO_2_–MSP,^28^ TiO_2_–waste plum,^29^ TiO_2_–corn cob,^25,30^ TiO_2_–chitosan,^31^ TiO_2_–wheat husks,^32^ TiO_2_–bamboo,^33^ TiO_2_–reed straw,^34^ g-C_3_N_4_/FeVO_4_–pine needles,^35^ ZrO_2_–wheat husks,^36^ g-C_3_N_4_–chestnut,^37^ TiO_2_–wood charcoal,^38^ and BiOX–biochar.^39^

We believe that it is high time to give a comprehensive review on the recent advancements of biochar-supported photocatalysts, in particular, on their syntheses, applications, functionality, and future prospects based on environmental sustainability. Therefore this review provides major scientific insight into BSPs synthesized from low-cost biochar combined with catalytic nanoparticles, summarizes their syntheses and physicochemical properties, discusses in depth the role of biochar in BSPs driving photocatalysis, highlights their applications in various fields, and provides useful suggestions for the future development of efficient and novel BSPs.

## 2. BSP synthesis methods

### 2.1 Sol–gel

**Fig. 2:**
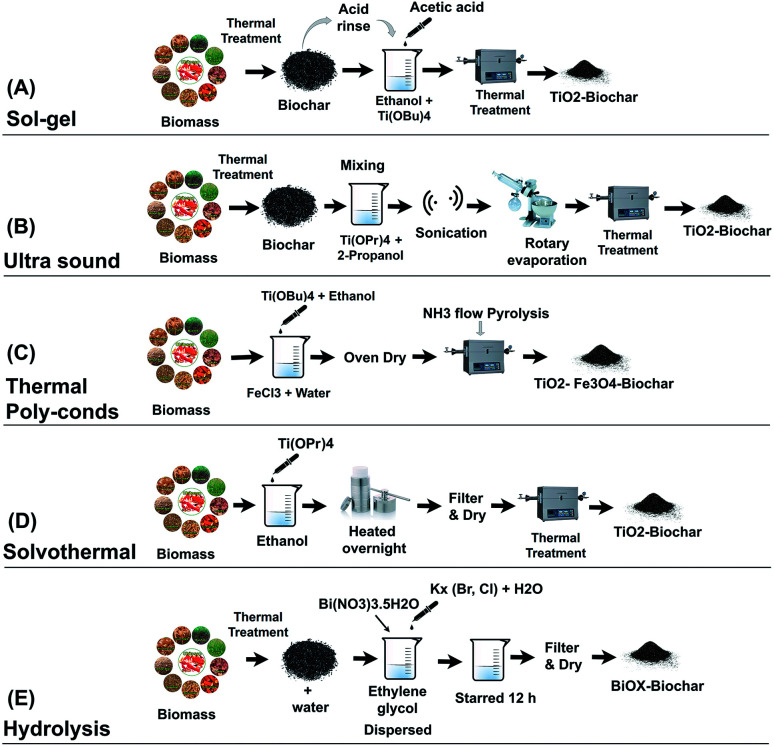
Schematic of BSP synthetic methods followed by interpretation of ref. 28, 31, 34 and 39.

Sol–gel is the most common method for synthesizing BSPs. In TiO_2_-BSP, granular-shaped anatase and/or rutile TiO_2_ is agglomerated on the biochar surface *via* a sol–gel synthetic method.^25^ The crystal phase structure, average size, and dispersion of TiO_2_ upon the biochar depends on the sol–gel decomposition temperature. Usually, calcination temperatures below 700 °C can create anatase TiO_2_ crystals. The dispersion of TiO_2_ and changes in biochar structure increase with the calcination temperature.^34^ At high temperatures above 700 °C, TiO_2_ starts forming the rutile crystalline structure and the percentages of rutile increase with the rising temperature.^40^ BSP synthesis through sol–gel methods shows significant alteration of oxygen-containing functional groups due to their involvement in TiO_2_ doping with biochar. A typical sol–gel method follows three sequential formation steps: (1) prepare biochar templates *via* thermal decomposition of biomass, (2) increase surface oxides and reduce pH of biochar *via* acid treatment, then deposit catalytic nanoparticles on the biochar surface, and finally (3) calcine the obtained biochar-laden catalytic nanoparticles to give a stable structure.^25,34,41^ The schematic of the BSP synthetic process using the sol–gel method is illustrated in Fig. 2A. Recently, Zhang *et al.*^34^ synthesized an efficient TiO_2_–reed straw BSP using the sol–gel method for the photodegradation of sulfamethoxazole. They produced the biochar template by heating at a temperature of 500 °C for 6 h with a heating rate of 20 °C min^−1^. The obtained biochar was crushed and sieved through a 0.15 mm mesh, and rinsed with HCl acid (1 mol L^−1^) for 2.5 h to increase the number of surface oxides and reduce the pH at the point of zero charge. The acid-treated biochar was used as the precursor for TiO_2_ doping. Later, the precursor was immersed in 50 mL ethanol and 20 mL Ti(OBu)_4_ solution, and 2.5 mL acetic acid was added dropwise into that solution. The prepared mixture was stirred for 2 h at room temperature. Then, 2.5 mL of ultra-pure water (pH = 2) was added and stirring was continued for a further 4 h. After stirring the obtained mixture, it was dried in an oven at 105 °C. The dried sample of Ti(OBu)_4_-laden biomass was further calcined in a muffle furnace at different temperatures (300–500 °C) to obtain the TiO_2_–biochar.

### 2.2 Ultrasound

Ultrasound (US) is a very new technique for the synthesis of BSPs. Recently, Lisowski *et al.*^28^ followed a multistep synthetic process with ultrasound mechanisms for the preparation of TiO_2_–softwood pellets and TiO_2_–miscanthus straw pellet biochar. The BSPs obtained contain enormous cracked structures on the surface that confirm the formation of a mesoporous structure. Using US treatment, anatase TiO_2_ crystal structures formed on the BSPs, and the growth of the TiO_2_ crystal size and structure is significantly higher than that of BSPs formed by a non-US method. A local trapping state due to Ti^3+^ below the TiO_2_ conduction band reduces the band gap of TiO_2_, and therefore the obtained material shows visible light sensitivity. US methods can act as interfacial mediators to improve the visible light response of the photocatalyst. The schematic of the multistep process with US mechanisms for the synthesis of BSPs is illustrated in Fig. 2B. Firstly, biochar was prepared through a thermal decomposition process at 500–700 °C. Then, the obtained biochar was processed for further treatment by crushing, sieving, washing, and drying. The clean and dry biochar (500 g) was mixed with a titanium(iv) isopropoxide and propanol solution, and that mixture was ultrasonicated for 1 h. After sonication, the materials were extracted using a rotary vacuum evaporator assisted by sonication. The sonicated material was dried and further calcined at 400 °C to prepare the TiO_2_–biochar.

### 2.3 Thermal polycondensation

Thermal polycondensation is a simple and single-step heating synthesis of BSPs. Recently Liu *et al.*^37^ and our research group synthesized BSPs using a thermal polycondensation process. Liu *et al.* followed a multistep calcination process where biochar was obtained firstly from a thermal decomposition process. Then, the obtained biochar was blended with melamine in different ratios and ground. Later, the mixture of melamine–biochar was placed in a furnace and calcined at 300 °C for 2 h with a heating rate of 5 °C min^−1^ for the synthesis of BSPs. However, our research group produced an agar biochar-based catalyst (N–TiO_2_–Fe_3_O_4_–biochar) *via* a single-step thermal polycondensation process that is shown in Fig. 2C. The natural polysaccharide agar was used due to its thermally responsive sol–gel properties. At low temperatures (30–40 °C), agar formed a gel network, which is beneficial for doping with other elements.^42,43^ The nitrogen functional groups were activated on the catalyst *via* NH_3_ ambiance calcination. The obtained BSPs (N–TiO_2_–Fe_3_O_4_–biochar) had a significantly reduced band gap energy, and therefore, the material is able to perform excellent visible light photocatalysis for methylene blue degradation. It is well-known that the appropriate proportion of transition metal Fe- and N-doping with TiO_2_ nanoparticles can significantly reduce the band gap energy^44^ and improve visible light photocatalysis.^7,45,46^ Furthermore, the Fe_3_O_4_ of the composite can activate the magnetic properties of the BSPs, which is suitable for easy collection from the aqueous media.

### 2.4 Solvothermal

TiO_2_-aggregated hollow spheres supported by carbon structures of furfural, saccharose, or chitosan can be prepared *via* a solvothermal method. Mainly anatase and some rutile TiO_2_ crystal structures formed on the composites through solvothermal synthesis. The crystal size of the sphere increased after calcination. The typical solvothermal synthesis follows several sequential steps (Fig. 2D). Firstly, catalytic nanoparticles such as TiO_2_ are deposited on the biomass templates *via* mixing them in an ethanol solution. For example, 1 g of carbon supporting materials such as furfural,^47^ chitosan, or saccharose^31,48,49^ and 1 g of titanium(iv) isopropoxide are dissolved in 18 mL of ethanol. Secondly, the prepared solution is sealed into a Teflon-lined stainless steel autoclave followed by solvothermal treatment at ∼175 °C overnight. The resultant solid after treatment is collected by filtration, washed several times, and dried. Finally, that dry sample is calcined at 600–800 °C for 6 h to remove the inner materials and form TiO_2_ hollow spheres. Composites synthesised by solvothermal methods show a reduction in the band gap energy and an improvement in visible light photocatalysis.

### 2.5 Hydrolysis

Some researchers have reported the synthesis of BSPs using a simple hydrolysis process, and improvement in visible light sensitivity during photocatalysis of organic pollutants. Min *et al.*^39^ incorporated BiOX (X = Br or Cl) with biochar through a hydrolysis method for the degradation of methyl orange. The schematic for the hydrolysis process for the synthesis of BSPs is given in Fig. 2E. Firstly, 2 mmol Bi(NO_3_)_3_·5H_2_O was dispersed in 20 mL ethylene glycol at ambient temperature. Meanwhile, pre-prepared biochar was evenly dispersed into 20 mL pure water by sonication. Later, the biochar solution was added dropwise into the Bi(NO_3_)_3_·5H_2_O solution. Afterwards, a pre-prepared KX (Br, Cl) solution (2 mmol KX with 20 mL pure water) was poured slowly into the mixture. The prepared mixture was stirred for a further 12 h at ambient temperature. Finally, the solid residue of BiOX (Br, Cl)–biochar in the mixture was collected, washed, dried, and stored for further use. Except for the heating required for biochar production, further heating is not required for BSP synthesis by the hydrolysis method, which is energy-saving.

## 3. Role of biochar in BSPs and photocatalysis

### 3.1 Supporting nanoparticles

Biochar can give an excellent platform for supporting a large number of nanoparticles. Simple thermochemically converted biochar contains available surface functional groups (C=O, C–O, COOH, COO–, OH *etc.*) and an enormous pore structure. The surface functional groups of biochar can easily be reacted with other functional materials. Moreover, some metallic and non-metallic mineral-containing biomass produced by thermal conversion shows the existence of N, S, P, K, Ca, and Mg content in biochar,^50^ which can directly act as an adsorbent, catalyst, or catalyst support.^51^ A recent review article by Tan *et al.*^52^ stated the general overview of different nanoparticle incorporation with biochar templates, their synthesis, and applications for environmental pollution decontamination. The tuning of biochar with different nanoparticles improves biochar functionality and pollutant removal competency *via* adsorption and/or catalytic degradation. Nanoparticles can combine with biochar in two ways: (1) thermal decomposition of nanoparticle-loaded biomass, and (2) tuning nanoparticles with prepared biochar functional groups through chemical modification. For example, Yang *et al.*^53^ synthesized Fe_3_O_4_-deposited biochar *via* a single step pyrolysis of FeCl_3_-laden sawdust biochar. Initially, the FeCl_3_-laden biochar hydrolyzed to form Fe(OH)_3_ and FeO(OH) during the drying process. After subjecting the FeCl_3_ biomass to pyrolysis, the biomass components such as lignocellulose decomposed quickly into volatile components and biochar. Meanwhile, FeO(OH) was reduced to form Fe_3_O_4_ by the reducing agents H_2_, CO, and amorphous carbon which were produced by the pyrolysis reaction. The advantages of metal-laden biomass pyrolysis are: a single step heating process, simultaneously producing nanoparticle-loaded biochar, and metal compounds which can have some catalytic effect on biochar pyrolysis which improves bio-oil and biochar textural properties.^22^

In recent years, natural polysaccharides and their derivatives have received significant interest in the synthesis of biochar-based materials due to their availability, low toxicity, biocompatibility, biodegradability, and easy modifiability with various nanoparticles.^54^ In drug delivery systems, natural polysaccharides are commonly used for carrying nanoparticles.^55^ Among different natural polysaccharides, chitosan is the most widely used polysaccharide in different fields of research as well as for biochar production.^56^ The nanoparticles can incorporate with chitosan easily by beading, crosslinking, grafting, and surface impregnation.^57^ Chitosan is soluble in acidic water due to the protonation of NH_2_. Acid-soluble chitosan can turn back into a flock structure by adjusting the pH to 7.5–9 with NaOH due to deprotonation and insolubility of the polymer at natural pH.^56^ Using this mechanism, an excellent iron-based γ-Fe_2_O_3_ biochar has been synthesized from water hyacinths and applied for the removal of Cr(vi).^58^ Recently, our research group used agar for the synthesis of a N–TiO_2_–Fe_3_O_4_ doped photocatalyst. The gel matrix of agar at low temperature gives an excellent platform for supporting TiO_2_ and Fe_3_O_4_ nanoparticles. The enormous number of OH or NH functional groups in natural polysaccharides is easily tunable with the catalytic nanoparticles.

### 3.2 Increasing surface area and number of active sites

Usually, catalytic nanoparticles have a small surface area. When nanoparticles are dispersed on the biochar surface, the synthesized material (BSP) surface area can have an increased surface area compared to the bare nanoparticles. However, the overall surface area of biochar may decrease due to aggregation of metallic nanoparticles.^37^ Nanoparticles can homogeneously disperse on the biochar surface *via* suitable modification processes. Well-dispersed surface nanoparticles can improve light scattering and increase the number of active sites, which enhances photodegradation of organic pollutants. Lisowski *et al.*,^28^ Balu *et al.*,^59^ and Wang *et al.*^60^ stated that the larger surface area and adequate number of active sites of TiO_2_–biochar composites can lead to the materials exhibiting greater photocatalytic effects due to the presence of more photocatalytic surface-active site centers. Moreover, biochar templates can provide an aromatic carbon structure and different hydrophilic and hydrophobic surface functional groups that can favor adsorption of organic pollutants *via* π–π stacking, hydrogen bonding, and/or electrostatic interactions. The higher adsorption competencies of the catalyst can facilitate the photodegradation. Nawi *et al.*^61^ reported that the synergies of adsorption–photodegradation of aqueous phenol by TiO_2_–chitosan can increase the degradation competency and reduce charge carrier recombination. Similar mechanisms were also reported by Zhang *et al.*^34^ for photocatalytic degradation of sulfamethoxazole by TiO_2_–reed straw biochar. Biochar-supported TiO_2_ prevented e^−^/h^+^ recombination and increased sulfamethoxazole photodegradation from 58.47% (commercial TiO_2_) to 91.27%.

### 3.3 Shuttling electrons

Some amorphous biochars have semiconductor characteristics, which helps to improve the reactivity when they are incorporated with nanoparticles *via* transferring electrons. A large number of studies have provided significant evidence about biochar electrical conductivity in different systems.^62^ Biochars facilitated the electron transformation from a bulk chemical electron donor to the receptor.^63,64^ Biochar surface redox-active moieties mediate the mechanisms of electron shuttling between different reactants. The condensed aromatic ring structure of biochar can promote the electron transfer process.^62,63^ Wu *et al.*^65^ stated that Fe_3_O_4_ nanoparticle-incorporated carbonaceous gel networks derived from watermelon biomass allow continuous and stable shuttling of electrons and electrolyte ions to the electrode surface. The excellent electrochemical behavior of Ag nanoparticle-covered waste biochar was also reported by Yao and Wu.^66^ In the TiO_2_–biochar or any BSP, the dense aromatic structure of biochar was able to shuttle electrons from the activation sites to the acceptors such as organic pollutants. This electron shuttling can reduce the quick recombination of e^−^/h^+^ pairs and enhance the photodegradation of pollutants.

### 3.4 Electron reservoir

**Fig. 3:**
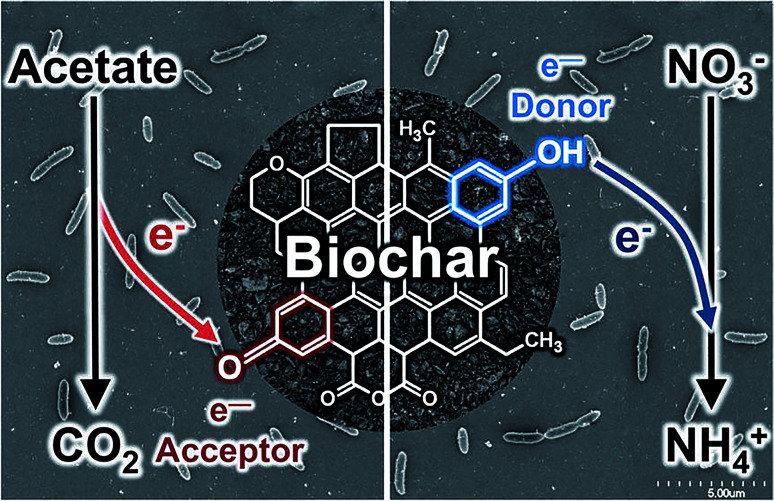
Electron storage mechanisms of biochar *via* quinone moiety electron acceptance and donation. Reproduced with permission from ref. 68.

Biochar also can act as an electron reservoir. It is important to note that the electron storage and shuttling mechanisms of biochar are different processes. The electron shuttling process requires the conductive domain of black carbon (graphene) or the dense aromatic structure of biochar, while electron storage is the function of oxidation and reduction of biochar quinone moieties. A small number of studies have been conducted to investigate the electron reservoir potential of biochar. Klüpfel *et al.*^67^ reported that plant-based biochar has the capacity to store electrons up to 2 mmol e^−^ per g. However, the electron storage capacity (ESC) of biochar depends on the types of biomass and the pyrolysis conditions. Biochar synthesized by intermediate to high heat temperatures shows a higher electron storage capacity *via* electron donation–acceptance. The electron donation and acceptance mechanisms by quinone moieties are illustrated in Fig. 3. Similar to humic acid, biochar has an enormous quinone content and can act as both electron donor and acceptor. Further, Saquing *et al.*^68^ found that wood-driven black carbon biochar has 0.85 and 0.87 mmol e^−^ per g ESC in the acetate oxidation and nitrate reduction system. The electron sink capacity of biochar can be investigated by electron balance calculations. Saquing *et al.* showed that a portion of the electrons were missing during ethanol oxidation by *Geobacter metallireducens*, indicating the electron sink behaviour by the biochar. Xu *et al.*^69^ synthesized TiO_2_–carbon nanotubes (CNTs) and stated that CNTs can act as electron reservoirs during the photodegradation of benzene and methyl orange. In TiO_2_–biochar composites, carbon can act as an electron reservoir that conducts away e^−^ from the e^−^/h^+^ pair and activated TiO_2_ during photocatalysis. Acting as an electron reservoir increases the efficiency of charge separation from TiO_2_ and hinders the e^−^/h^+^ pair recombination.^2^

### 3.5 Improving charge separation

The charge separation due to the e^−^/h^+^ pair is one of the important aspects of photocatalysis. Usually, bare semiconductor photocatalysts are activated by charge separation after light irradiation. However, they could recombine sooner with each other and achieve deactivation. In that case, a secondary component may maintain or even strengthen the activated state of the semiconductor photocatalyst, shown in reactions (R1)–(R4) following the interpretation of Shi.^70^

R1 P → P*

R2 P* → P

R3 P–C → P*–C

The single catalyst is activated after light irradiation (R1), while without any secondary element, the activated catalyst achieves deactivation again (R2). However, after a secondary element is incorporated into the catalyst, the activated state of the catalyst is well-maintained. Combination leads to activation of the catalyst (P) by other catalysts or non-catalysts (C) (R3). This accelerates the catalytic kinetic reaction and in some special cases both components can be mutually activated (R4).

R4 P + C → P*/C*

In the BSP, electron shuttling from the catalytic nanoparticle to carbon significantly improves charge separation. The charge separation competency of the photocatalyst can be detected by photoluminescence (PL) spectroscopy and the photocurrent response. The lower PL spectrum intensity and the higher photocurrent density indicates the higher efficiency of the electron–hole pair separation.^28,39^ Lisowski *et al.*^28^ synthesized TiO_2_–biochar through a simple ultrasound sonication process and indicated that the charge separation significantly increases when biochar is incorporated with TiO_2_. The formation of heterojunctions between the TiO_2_ and biochar templates is responsible for the photoinduced charge separation. The similar increase in charge separation due to Fe and FeVO_4_ heterojunctions in the g-C_3_N_4_–FeVO_4_–Fe@NH_2_–biochar were reported by Kumar *et al.*^35^ Further, Zhang *et al.* synthesized TiO_2_@SiO_2_ wrapped in a GO layer and state that the thin GO layer improves the charge separation. However, the thick layer of GO negatively affects the charge separation due to adsorbance or scattering of photon energy, and therefore the photocatalytic activity decreases. The similar masking effects of a thick carbon layer on photocatalysis also reported by Wu *et al.*^33^

### 3.6 Reducing band gap energy

**Fig. 4:**
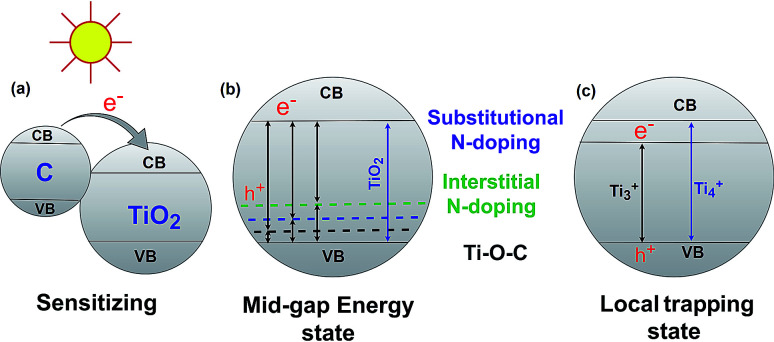
Reduction of the TiO_2_–BSP band gap energy *via* sensitizing (a), forming a mid-gap energy state (b) and forming a local trapping state (c).

The band gap energy of semiconductor photocatalysts can be reduced by combining them with biochar. There are three major ways that have been reported in the literature for reducing the TiO_2_–BSP band gap: (1) sensitize the photocatalytic nanoparticle with another compound, (2) create a mid-gap energy state *via* doping non-metals such as N, S, C, O *etc.*, and (3) formation of a local trapping state below the TiO_2_ conduction band. Usually, composites with small band gaps are used to sensitize the larger band gap semiconductor photocatalyst (Fig. 4a). Carbon can act as a sensitizer when doping with TiO_2_. The larger band gap of TiO_2_ can be sensitized by carbon doping,^71^ and therefore, the synthesized composite light sensitivity shifts from the UV region to the visible light region. For efficient sensitization, the conduction band of the sensitizer material should be placed higher than the conduction band of TiO_2_.^72^ Another important aspect of the TiO_2_–BSP is the energy level of any impurities which form Ti–O–C bonds.^73,74^ This can create a delocalized state in the band gap of TiO_2_ without promoting charge carrier recombination.^7^ Furthermore, the incorporation of non-metals N or S with the TiO_2_–biochar significantly reduces the band gap of the composite by forming a mid-gap energy state. The excellent N- and S-doped biochar-based TiO_2_ semiconductor photocatalyst has been synthesized by Matos *et al.*^29^ and Bandosz *et al.*^75^ Due to N- and S-doping, the photocatalytic activity of the TiO_2_-composite increases up to 2 and 5 times more than the bare TiO_2_ photocatalyst. However, the photocatalytic activity of the composites is largely influenced by the texture and the content of S and N in the BSP.^29,75^ N- or S-doped BSPs not only reduce the energy gap of TiO_2_ but also act as electron shuttles from the π-orbital of the N/S-doped biochar to the conduction band of TiO_2_. The general procedures for reducing the band gap energy level by C-, substitutional N-, and interstitial N-doping in TiO_2_–biochar are illustrated in Fig. 4b. An excellent review article by Ansari *et al.*^76^ describes in detail the reduction of the band gap by N-doping semiconductor photocatalysts. The local trapping state below TiO_2_ can also be produced in TiO_2_–biochar as shown in Fig. 4c. Lisowski *et al.*^28^ reported that a TiO_2_–biochar synthesized by an ultrasound method contained a local trapping state corresponding to Ti^3+^, which reduced the band gap of the BSP as well as enhancing the photocatalytic redox reaction.

## 4. Applications of BSPs

Many excellent new technologies can be understood *via* applications of BSPs, extending the existing wide range of photocatalytic nanoparticle applications. The most prominent application of BSPs is in the degradation of various hazardous organic pollutants in air and waterways. Furthermore, these BSPs can also be applied as film electrodes in solar cells, and as photocatalytic H_2_ generators in water splitting. The list of BSPs which have been synthesized and their applications in various fields are summarized in Table 1. Organic pollutants whose photodegradation by BSPs have been published at least once include phenol,^28,77^ bisphenol A,^38^ methylene blue,^29,33,37,75^ safranin T,^41^ anthraquinone,^32^ malic acid,^59^ sulfamethoxazole,^25,30,34^ methyl paraben,^35^ 2-chlorophenol,^35^ Reactive Yellow 39,^36^ methyl orange^39^ in the liquid phase, and methanol in the gas phase.^28^ Among the listed pollutants, methylene blue is the most commonly studied pollutant followed by sulfamethoxazole and phenol. The photodegradation of methylene blue by various BSPs ranges from 38% of 4.8 ppm to 100% of 400 ppm, and the degradation time varies from 1.5 h to 4 h. Phenol degradation capacity ranges from 64% to 75% of 50 ppm within 4 h, and sulfamethoxazole degradation capacity is ∼90% of 10 ppm within 3 h. The intermediate product analysis during photodegradation can give a clear understanding of the degradation pathway and evidence of mineralization. For example, the quantification of malic acid and methanol mineralization in water and air media by TiO_2_–guanidine–(Ni,Co)–Fe_2_O_4_ (ref. 59) and TiO_2_–SWP700,^28^ respectively give a clear understanding of the pollutant photodegradation process. It shows that the overall conversion rate of malic acid and methanol was 60% and 88%, respectively. Similarly, methyl paraben, 2-chlorophenol, and sulfamethoxazole photodegradation pathways were investigated by Kumar *et al.*^35^ and Zhang *et al.*,^34^ respectively. Biochar-supported photocatalysts are also applicable for the degradation of pollutants in the gaseous phase. Lisowski *et al.*^28^ passed a mixture of methanol and air (0.9% + 99.1% vol) at a flow rate of 25 cm^3^ min^−1^ through an author-designed BSP-containing photo-reactor. The reaction output products were measured online by gas chromatography and offline by GC-MS under conditions both with and without UV light illumination. The UV illumination and presence of TiO_2_–SWP700 significantly mineralized methanol into CO_2_ and methyl formate. Besides, organic pollutant degradation, BSPs also show good performance as film electrodes in solar cells and as H_2_ producers in water splitting. Matos *et al.*^31^ synthesized TiO_2_–C hollow spheres *via* a solvothermal and post-calcination process of TiO_2_–chitosan/furfural/saccharose precursors. The obtained BSPs were successfully applied as film electrodes in a solar cell under visible light catalysis. Furthermore, Matos *et al.* synthesized^78^ Au–TiO_2_ biochar through multi-step heating and slurry methods and applied them for H_2_ production from water. The author states that the biochar-supported Au–TiO_2_ performs 3 times better photocatalysis than the commercial semiconductor photocatalyst and the O functional groups of biochar enhance the photocatalytic H_2_ production. It also worth mentioning that BSPs possess high recyclability during organic pollutant photodegradation. In most cases, their organic removal capacity decreased only 2–10% after recycling the BSPs 3–6 times (Table 1), which indicates the higher stability, recyclability and cost effectiveness of the composites.

**Table 1:** Synthetic processes and applications of various biochar-supported photocatalysts

Name of catalyst	Biomass	Metal	Application	Initial pollutant concentration	Removal capacity (Re) and recycle capacity (Rc)	Time	Process of removal	BSP synthetic process	Ref.
TiO_2_–biomass	Starbon	Ti	Phenol photodegradation	150 mL – 50 ppm	Re: 75%	4 h	Adsorption and photodegradation	Ultrasound sonication	77
TiO_2_–SWP700	Soft wood pellets	Ti	Phenol photodegradation	150 mL – 50 ppm	Re: 64%	4 h	Adsorption and UV + Vis light irradiation	Ultrasound sonication	28
					Rc: (∼59–67%)/5 times				
TiO_2_–wood charcoal	Pine wood	Ti	Bisphenol A photodegradation	50 mL – 20 ppm	Re: 80%	18 h	Adsorption and photodegradation	Dip-sol-gel method	38
N–TiO_2_–C	Waste plum stones	Ti	Methylene blue photodegradation	125 mL – 25 ppm	Re: 100%	3 h	Adsorption and photodegradation	Multi-step pyrolysis and slurry method	29
TiO_2_–bamboo	Bamboo	Ti	Methylene blue photodegradation	200 mL – 30 ppm	Re: 99%	1.5 h	Adsorption and UV + Vis light irradiation	Hydrothermal carbonization and sol–gel methods	33
					Rc: ∼100%/4 times				
g-C_3_N_4_–biochar	Chestnut leaf biomass		Methylene blue photodegradation	20 mL – 4.8 ppm	Re: 38%	4 h	Photodegradation	Multi-step thermal polycondensation	37
S-activated carbons	Activated carbon		Methylene blue photodegradation	125 mL – 25 ppm	Re: 100%	4 h	Adsorption and photodegradation	Multi-step heating process	75
N–TiO_2_–Fe_3_O_4_–biochar	Agar	Fe, Ti	Methylene blue photodegradation	10 mL – 400 ppm	Re: 100%	3 h	Adsorption, photodegradation, and Fenton-like degradation	Single-step thermal polycondensation	Our study
					Rc: (89–100%)/5 times				
TiO_2_-coated biochar	Ramie bar	Ti	Safranin T photodegradation		Re: 231.9 mg g^−1^	2 h	Adsorption and photodegradation	Sol–gel method	41
					Rc: (167–222 mg g^−1^)/6 times				
TiO_2_–sludge and wheat husks	Sludge and wheat husks	Ti	Reactive Blue 69 photodegradation	20 ppm/1.5 g L^−1^ dose	98%	1.3 h	Ultrasound irradiation	Sol–gel method	32
TiO_2_–guanidine–(Ni,Co)–Fe_2_O_4_	Biomass	Ti, Ni, Co, Fe	Malic acid photodegradation	0.5 mmol/50 mL water	Re: conversion = 60%, acetic acid = 10%, formic acid = 77%, oxalic acid = 7%, CO_2_ = 8%	1.5 h	Photodegradation	Hydrothermal process	59
TiO_2_–corn cob	Corn cob	Ti	Sulfamethoxazole photodegradation	100 mL – 10 ppm	Re: 90%	3 h	Adsorption and UV light irradiation	Sol–gel method	25
					Rc: (90–92%)/3 times				
TiO_2_–reed straw	Reed straw	Ti	Sulfamethoxazole photodegradation	160 mL – 10 ppm	Re: 91%	3 h	Adsorption and UV light irradiation	Sol–gel method	34
					Rc: (86–91%)/5 times				
g-C_3_N_4_–FeVO_4_–Fe@NH_2_–biochar	Pine needles	Fe, V	Methyl paraben (MeP) and 2-chlorophenol (2-CP) photodegradation	100 mL – 20 ppm	Re: 98.4% of MeP	1.5 h	Adsorption, photocatalysis, and photo-ozonation	Multi-step thermal treatment, acid treatment, and ammonia treatment	35
					Rc: (97–98%)/6 times				
					Re: 90.7% of 2-CP				
					Rc: (89–91%)/6 times				
ZrO_2_–sludge and wheat husks	Sludge and wheat husks	Zr	Reactive Yellow 39 photodegradation	20 ppm/1.5 g L^−1^	Re: 98%	1.2 h	Ultrasound irradiation	Modified sonochemical and sol–gel method	36
BiOX (X = Cl or Br)–biochar		Bi	Methyl orange photodegradation	50 mL (0.03 mM)	Re: 10% BiOBr = 81%	2.5	Photodegradation	Hydrolysis method	39
					Re: 5% BiOCl = 38%				
TiO_2_–SWP700	Soft wood pellets	Ti	Methanol oxidation	(0.9% + 99.1%) phenol + air-flow rate of 25 cm^3^ min^−1^	Re: conversion = 88%, CO_2_ = 20%, methyl formate yield = 88%		UV + Vis light irradiation	Ultrasound sonication	28
TiO_2_–chitosan	Chitosan	Ti	Film electrodes					Solvothermal	31
Au–TiO_2_/AC	Waste plum stones	Au, Ti	H_2_ production		−22.5 mM at visible light, and −33 at UV light		UV light irradiation	Multi-step heating and slurry method	78

## 5. BSP-driven photodegradation mechanisms

### 5.1 Adsorption

**Fig. 5:**
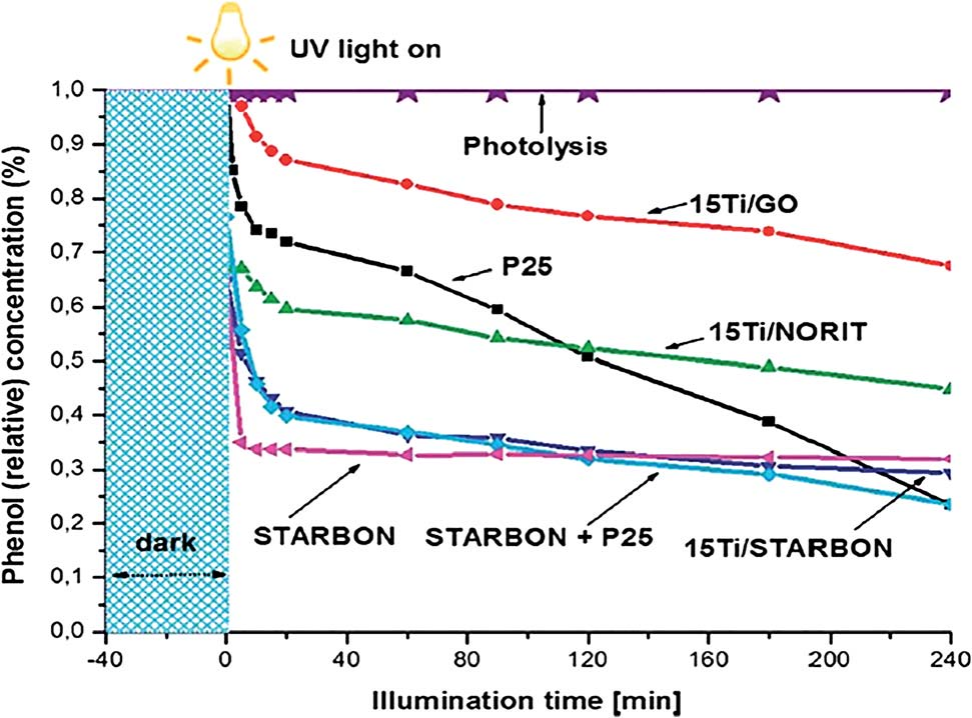
Photocatalytic degradation of phenol by different photocatalysts. Reproduced with permission from ref. 77.

Incorporating catalytic nanoparticles with biochar can increase the BSP affinity for adsorbing a variety of organic pollutants in an aqueous medium. A higher adsorption potentiality of BSPs enhances photodegradation of organic pollutants to a greater extent than bare nanoparticles.^33^ Colmenares *et al.*^77^ reported that similar Ti-doping with different biochar components significantly varies their pollutant removal competency. As shown in Fig. 5, 15Ti/GO, 15 Ti/Norit, and 15Ti/Starbon exhibit different phenol reduction competencies, even though a similar synthetic process and amount of Ti has been applied in their syntheses. The higher phenol reduction competency shown by 15Ti/Starbon is due to its higher adsorption competency, its hybrid interphase proximity, and the presence of a highly-crystalline anatase phase.

A similar example of higher photodegradation due to higher adsorption capacity of the composite was reported by Luo *et al.*^38^ The commercial P25 and author-synthesized TiO_2_–WC-450 exhibits a similar ratio of anatase and rutile TiO_2_ (80 : 20). However, TiO_2_–WC-450 exhibits 1.85 times higher removal of bisphenol A than P25, which was due to higher adsorption and mass transfer competency of biochar-supported TiO_2_. The biochar contents in the photocatalyst play a key role in the adsorption as well as in photodegradation. Due to surface adsorption capabilities, organic pollutants can deposit on the catalyst surface before transfer to the decomposition center. Therefore, the reactive oxygen species generated due to light illumination do not need to migrate so far from the activation site. However, some fractions of organic pollutants are adsorbed on the catalyst without coming into contact with TiO_2_. Those particles also undergo attack by ROS produced on the surface of TiO_2_. It is assumed that the ROS could diffuse over a sub-millimeter distance from the activation site to the reactant site located on the surface of the composites.^28^ The synergistic effect of adsorption and photodegradation give rise to an efficient photodegradation process.^34,60^ Kumar *et al.*^35^ reported that the simultaneous adsorption–photodegradation allow high and rapid degradation of methyl paraben (MeP) and 2-chlorophenol (2-CP) due to the immediate attack of ROS after being adsorbed onto the photocatalyst. However, adsorption followed by photodegradation reduces the degradation competency due to covering most of the surface active sites with the pollutants.^35^

### 5.2 Photodegradation

**Fig. 6:**
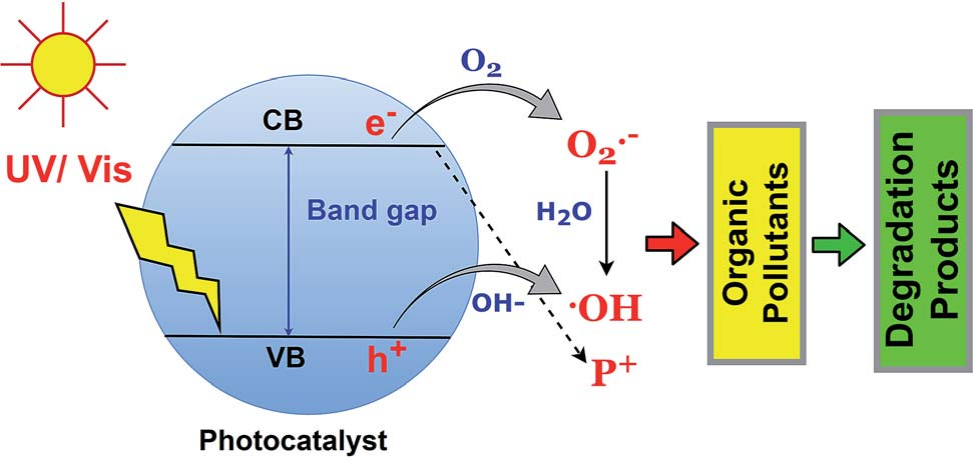
Fundamentals of organic pollutant photodegradation by ROS.

The photodegradation of organic pollutants using BSPs is the function of reactive oxygen species such as ˙OH and O_2_˙^−^, and h^+^. Identification of specific ROS functions during photocatalysis is essential to understand the photocatalytic redox reaction.^79^ Generally, the photocatalyst adsorbed a photon with an energy which is equal to or less than the band gap, and excited the electron in the valence band to the conduction band. Therefore, an electron–hole is created in the upper edge of the valence band and an electron occupies a phase on the lower edge of the conduction band. Later on, the h^+^ and e^−^ can either recombine or diffuse to the acceptor. The e_(CB)_^−^ and h_(VB)_^+^ can scavenge the –OH, O_2_, and H_2_O molecules in the liquid phase and successively produce ˙OH and O_2_˙^−^ species, which further react with organic pollutants leading to complete mineralization. The fundamentals of organic pollutant photodegradation by ROS are illustrated in Fig. 6. The role of specific ROS in various pollutant photodegradation using various BSPs is different. Recently, many researchers have been investigating the mechanisms of organic pollutant photodegradation using specific ROS identification and degradation product analysis. In most cases, ROS scavenging by different inhibitors and mass spectroscopic methods have been used.^33,80,81^ A comprehensive list of different ROS-function identification processes for organic pollutant photodegradation by BSPs is summarized in Table 2. Scavenger benzoquinone and TEMPOL for O_2_˙^−^, ammonium oxalate, triethanolamine, and EDTA 2Na for h^+^, and *tert*-butyl alcohol and isopropanol for ˙OH identification are very popular in ROS scavenging experiments. As shown in Table 2, the organic pollutants were degraded by the action of multiple ROS. The ranking was made based on specific ROS pollutant removal competencies determined by scavenging experiments. An excellent review article has recently been published with extensive discussion about the generation and detection process of ROS in photocatalysts.^79^ According to Nosaka and Nosaka, MCLA chemiluminescence, lucigenin chemiluminescence, 1270 nm emission and coumarin fluorescence probing are the most recommended methods for the detection of O_2_˙^−^, H_2_O_2_, O_2_, and ˙OH.

**Table 2:** Responsible ROS and their function identification processes in different organic pollutant photocatalysis by BSPs

Name of catalyst	Pollutants	ROS responsible for degradation	ROS function identification process	Ref.
TiO_2_–SWP700	Phenol	All	Scavenged by benzoquinone for O_2_˙^−^, ammonium oxalate for h^+^, and *tert*-butylalcohol for ˙OH	28
		Ranking: h^+^ > O_2_˙^−^ > ˙OH		
TiO_2_–bamboo	Methylene blue	All	Scavenged by benzoquinone for O_2_˙^−^, ammonium oxalate for h^+^, and *tert*-butylalcohol for ˙OH	33
		Ranking: O_2_˙^−^ > ˙OH > h^+^		
g-C_3_N_4_–biochar	Methylene blue	O_2_˙^−^ and h^+^	Scavenged by benzoquinone for O_2_˙^−^, triethanolamine for h^+^, *tert*-butylalcohol for ˙OH, and sodium azide for O_2_	37
		Ranking: O_2_˙^−^ > h^+^		
BiOX (X = Cl or Br)–biochar	Methyl orange	All	Scavenged by benzoquinone for O_2_˙^−^, ethylene diaminetetraacetic acid disodium salt (EDTA 2Na) for h^+^, and isopropanol for ˙OH	39
		Ranking: O_2_˙^−^ > h^+^ > ˙OH		
g-C_3_N_4_–FeVO_4_–Fe@NH_2_–biochar	Methyl paraben (MeP) and 2-chlorophenol (2-CP)	All	Scavenged by 4-hydroxy-2,2,6,6-tetramethylpiperidinyloxy (TEMPOL) for O_2_˙^−^, ethylene diaminetetraacetic acid disodium salt (EDTA 2Na) for h^+^, and *tert*-butylalcohol for ˙OH	35
		Ranking: ˙OH > O_2_˙^−^ > h^+^		
TiO_2_–reed straw	Sulfamethoxazole	˙OH and O_2_˙^−^	HPLC-PDA chromatograms	34

### 5.3 Ozonization

Ozonization during photocatalytic degradation of organic pollutants by BSPs influences reaction rate and speed. The combined mechanisms of adsorption, photodegradation, and ozonization enhance organic pollutant removal competencies to a greater extent.^35^ During the combined treatment, ozone leads to the production of ˙OH and H_2_O_2_ due to exposure to photon energy. Meanwhile, the electron which is unable to recombine due to the heterojunction in the BSP, substantially increases the production of O_2_˙^−^ and ˙OH by reaction with O_2_ and H_2_O_2_. Therefore, the reaction rate between ROS and pollutants increases, which results in a rapid and high reduction of pollutants. Furthermore, Fe-doped BSPs can give Fenton-like reaction competency. Our synthesized biochar-supported photocatalyst (N–TiO_2_–Fe_3_O_4_–biochar) exhibited very high methylene blue reduction under visible light irradiation due to injecting H_2_O_2_ in the reaction system. Similarly, graphene oxide-encapsulated Fe_3_O_4_–TiO_2_ spheres synthesized by Yang *et al.*,^8^ shows good Fenton-like degradation competency of methylene blue under visible light irradiation as a result of rapid redox reactions between Fe^2+^ and Fe^3+^.

Finally, it is also required to mention that the dose of organic pollutants and catalyst has a significant influence on the photodegradation process. Unlike adsorption of pollutants, photodegradation and simultaneous adsorption–photodegradation do not proportionally increase with the concentration of catalyst dose. Therefore, a specific dose of catalyst or pollutants may require determining to achieve optimum pollutant degradation. High concentrations of pollutants can prevent the light being able to reach the reactant photocatalyst. Therefore, the production of ROS can be reduced. Similarly, the dose of a catalyst has an optimum limit to reach maximum photocatalysis. An excessive photocatalyst dose can cause light scattering, which hinders the specific activity of the photocatalyst.^82,83^ Moreover, the number of active sites on the reactants can be reduced due to agglomeration of homogeneous particles at a high dosage.^84^ Therefore, an optimum dose of BSPs and pollutants should be measured to achieve optimum performance of the photocatalyst.

## 6. Conclusion and outlook

This review gives a detailed overview of the recent progress in the field of BSPs, summarized their synthetic processes and physicochemical characteristics, explicitly discussed the biochar role in the BSP, and pollutant photodegradation. It has revealed that the incorporation of transition metals/catalytic nanoparticles and/or non-metals with biochar templates are effective techniques to produce high-performance semiconductor photocatalysts. The catalytic nanoparticles can easily be incorporated with biochar through sol–gel, solvothermal, ultrasound, thermal polycondensation, and hydrolysis methods. The photocatalytic performance of BSPs significantly increases due to the unique physicochemical properties of biochar. Biochar can support a large number of nanoparticles, increase the number of active sites on the photocatalyst, act as an electron reservoir which conducts away the electron from the e^−^/h^+^, shuttle electrons through a graphene-like skeleton, improve charge separation, and reduce band gap energy by carbon or other non-metal doping with catalytic nanoparticles. Unlike bare catalytic nanoparticles, BSP adsorption capacity during photocatalysis enhances photodegradation performance. Simultaneous adsorption and photodegradation perform rapid and higher organic pollutant degradation due to the immediate attack of nearby ROS. Moreover, BSPs are more convenient to recover from aqueous solution than bare CNPs. Up until now, BSPs have mainly been applied for the photodegradation of organic pollutants in the aqueous phase. However, only a small portion of research focuses on BSP applications for clean energy production, air pollutant treatment, and anti-bacterial sterilization. It has already been proven that BSPs are an effective material for photocatalytic applications due to their cost-effectiveness, sustainability, and higher visible light photoactivity. However, there are still a number of aspects that need to be considered for the future development of BSPs.

• Prior to the synthesis of BSPs, it is worth considering the cost-effectiveness and the availability of natural biomass, a facile synthetic process to incorporate catalytic nanoparticles, and an efficient modification technique to enhance optical properties in the visible light region.

• Applying a wide variety of biomass and nanoparticles in the synthesis of BSPs and examining the synergistic effects of biochar and catalytic nanoparticle incorporation.

• The key controlling factors that stimulate BSP performance characteristics should be investigated such as heating operating conditions, selection and ratio of biomass and nanoparticles, and incorporation processes.

• Various hazardous waste materials such as sewage sludge, manure, and various organic and inorganic municipal waste materials can be used in the synthesis of BSPs. The waste material contains various catalytic nanoparticles that could improve the BSP photoresponse.^85^ Furthermore, natural polysaccharides have unique physicochemical properties such as formation of gels, flocking, and complete dissolution in water, which are beneficial to the incorporation of various compounds. This is a novel, sustainable and environmentally friendly way to manage municipal waste.

• Introduction of BSPs in many other photocatalytic applications and investigation of the photocatalysis mechanisms and performance.

## Conflicts of interest

There are no conflicts to declare.

## Supplementary Material
